# Supplementary material for: Integrated diversity and network analyses reveal drivers of microbiome dynamics
Source: mSystems. 2025 Sep 15;10(10):e00564-25. doi: 10.1128/msystems.00564-25 (PMC12542658; doi:10.1128/msystems.00564-25)
Supplement: Supplemental figures — Fig. S1 to S16. [file msystems.00564-25-s0001.pdf]

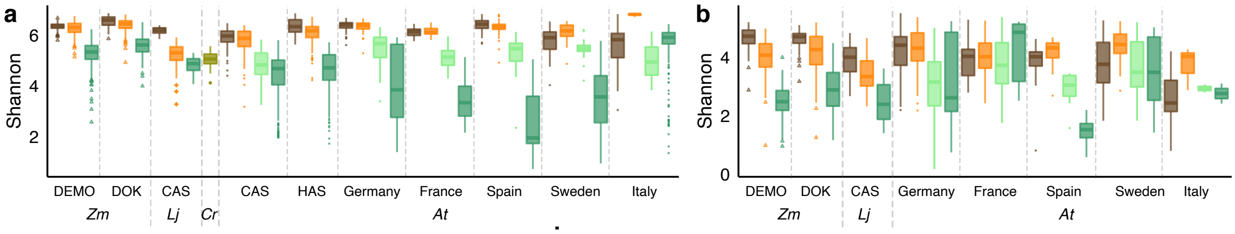

Figure S1 Alpha-diversity of plant- and alga-associated microbiota. Average Shannon indices of 999 times rarefaction and calculation were shown for bacterial (**a**) and fungal (**b**) samples under each condition. At: *Arabidopsis thaliana;* Zm: *Zea mays*; Lj: *Lotus japonicus;* Cr*: Chlamydomonas reinhardtii;* CAS*:* Cologne Agricultural Soil.

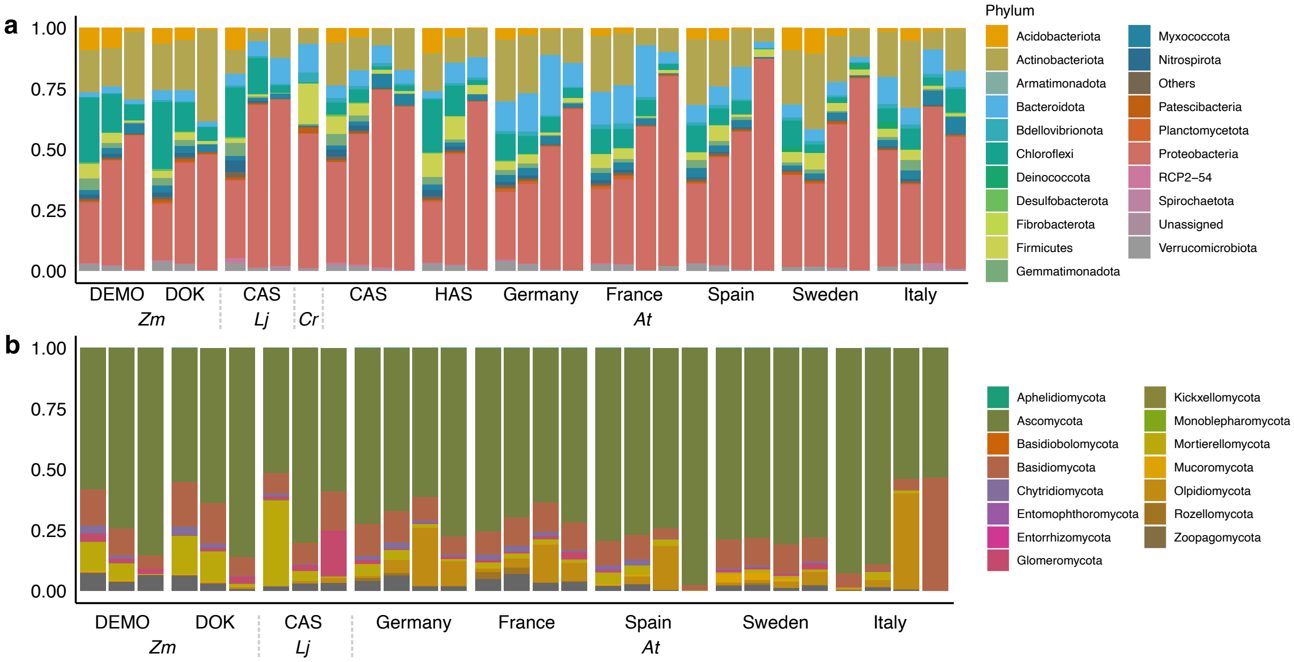

Figure S2 Taxonomic profiling of community structure at the phylum rank. The most abundant (with aRA > 0.1%) bacterial (**a**) and fungal (**b**) phyla are shown, and samples from different host species, soil types and compartments are compared here. At: *Arabidopsis thaliana;* Zm: *Zea mays*; Lj: *Lotus japonicus;* Cr*: Chlamydomonas reinhardtii;* CAS*:* Cologne Agricultural Soil.

Supplementary Figures

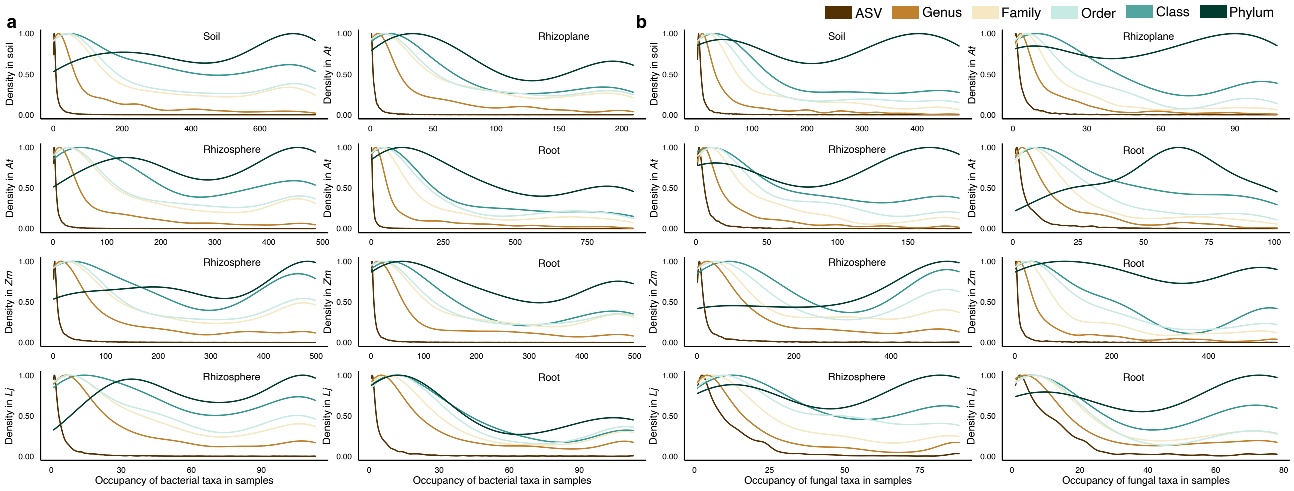

Figure S3 Density plots for microbial occupancies in different compartments and host species at multiple taxonomic levels. Bacterial (**a**) and fungal (**b**) microbes at each taxonomic levels, from ASV to phylum, are shown here. *At*: *Arabidopsis thaliana*; *Zm*: *Zea mays*; *Lj*: *Lotus japonicus*.

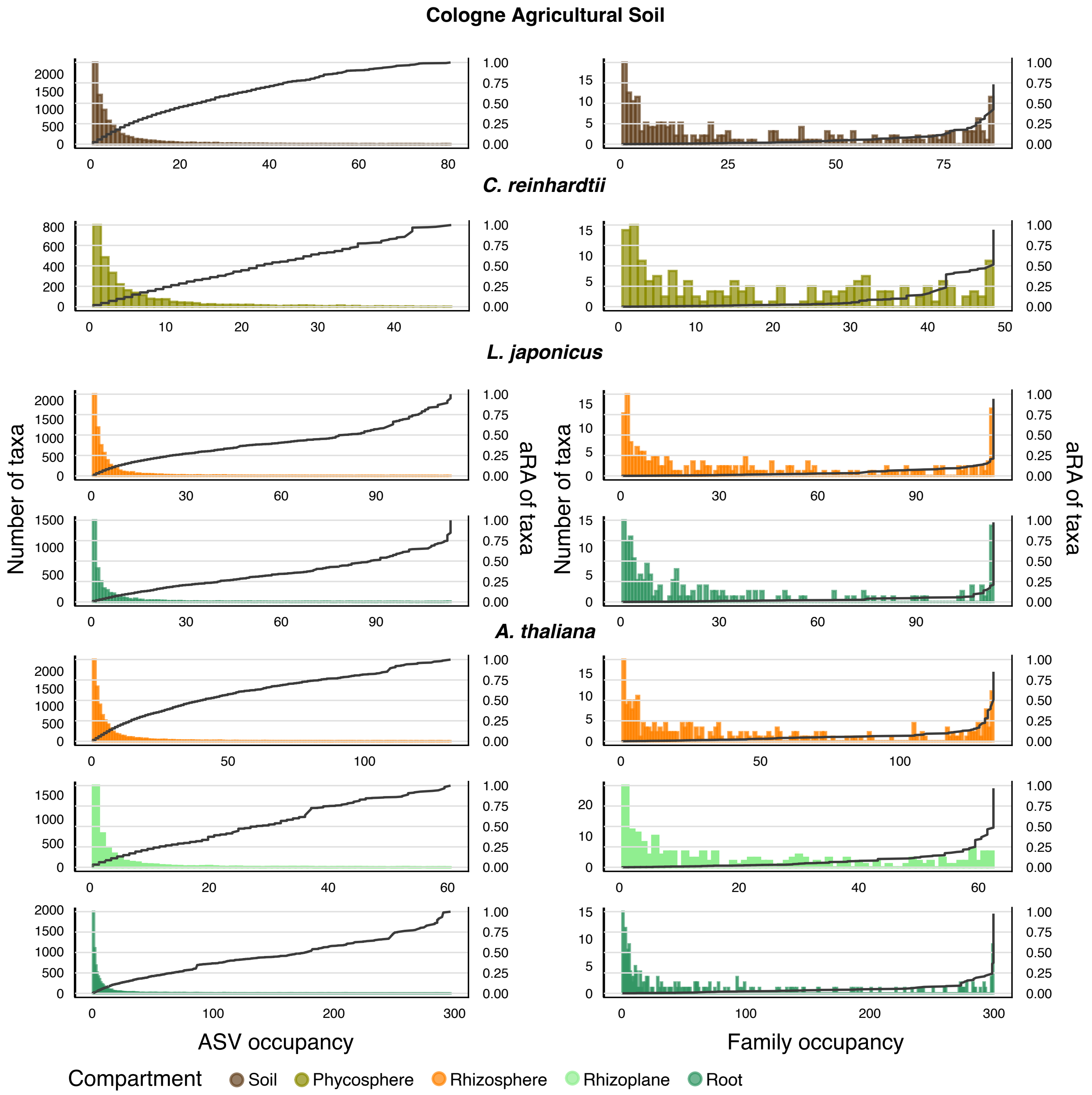

Figure S4 Occupancy and aRA of Cologne Agricultural Soil associated microbiota at the ASV and family levels. Within each condition (host species and compartment), no enrichment of prevalent composition at the ASV level. While at the family level, an increasing number of taxa with high occupancy was found, and they comprised a large portion of aRA, shown by the steep climbing of the black curve in the end. Left y-axis shows the number of taxa in bar plots colored by compartments and right y-axis shows the aRA indicated by the black line. aRA: accumulated relative abundance.

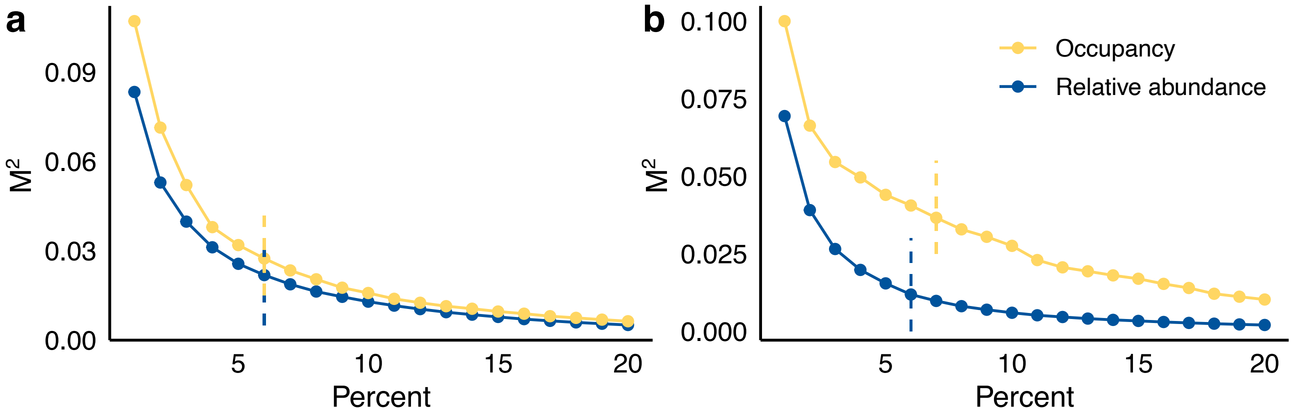

Figure S5 Procrustes distance between the diversity calculated from full and examined subset community members. Subset members of bacterial (**a**) and fungal (**b**) communities were chosen and assessed for both RA and occupancy with thresholds from 1% to 20%. Vertical dash lines indicate the determined threshold for representative ASVs. M^2: Procrustes distance.

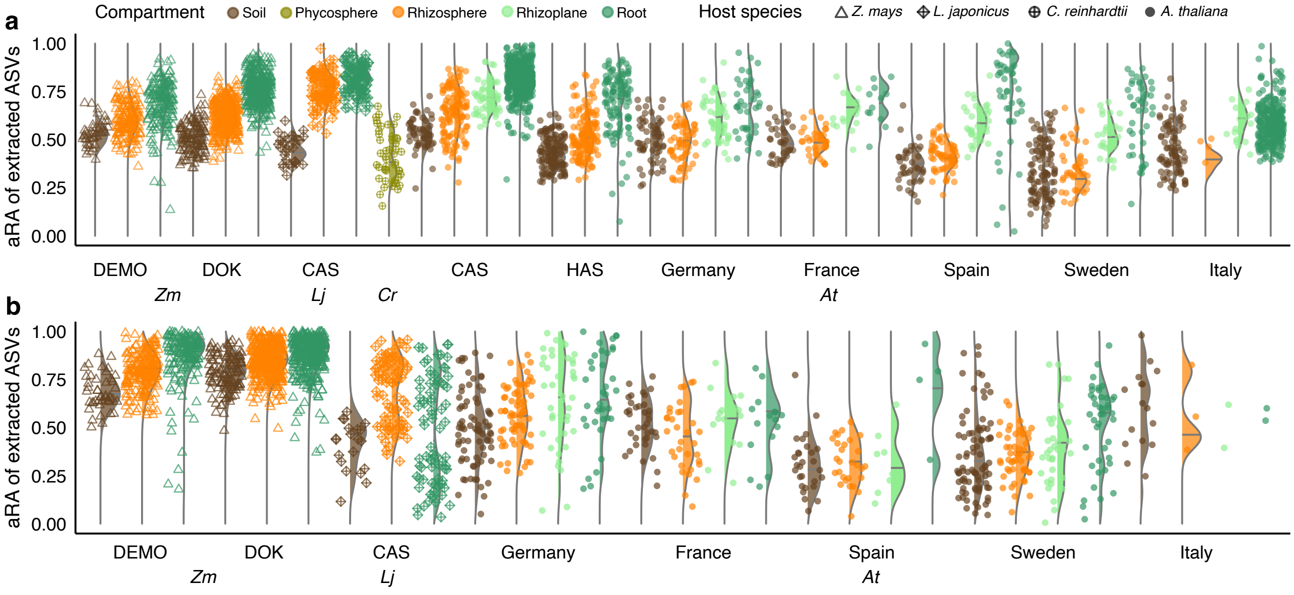

Figure S6 Aggregated relative abundance of the representative ASVs. Bacterial (**a**) and fungal (**b**) communities from different compartments, host species and soil types are compared and demonstrated here. At: *Arabidopsis thaliana;* Zm: *Zea mays*; Lj: *Lotus japonicus;* Cr*:* *Chlamydomonas reinhardtii;* CAS: Cologne Agricultural Soil.

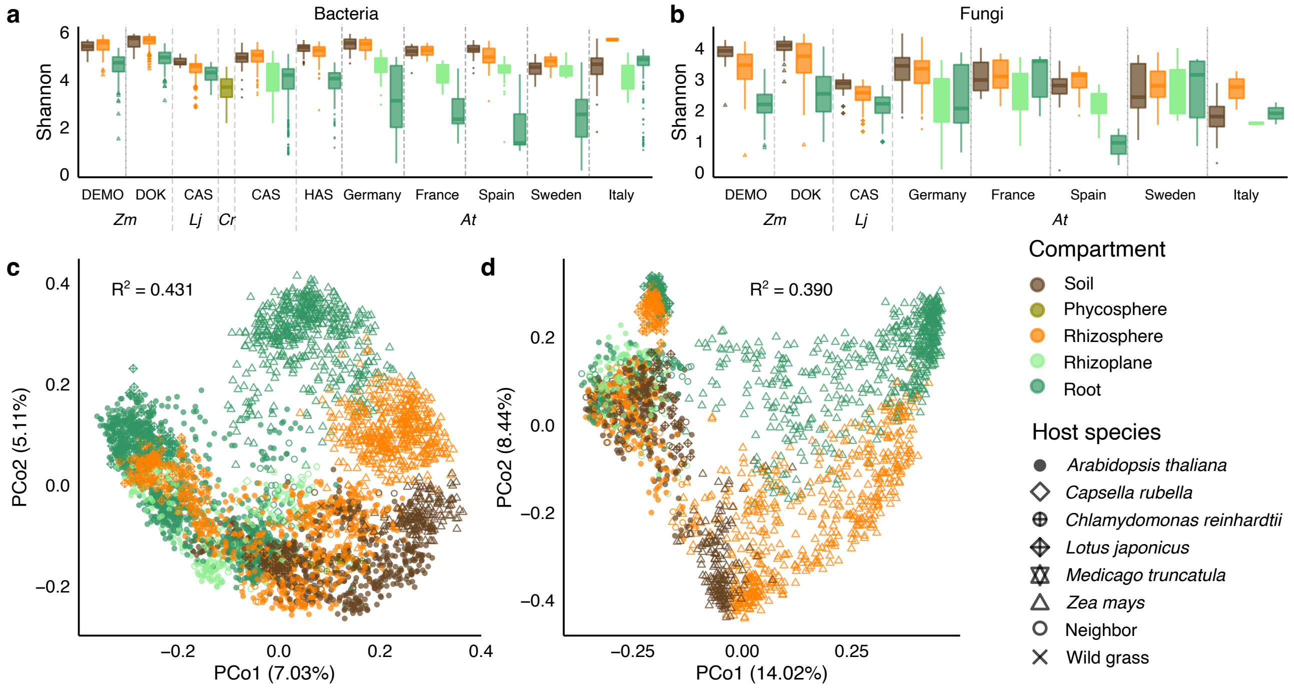

Figure S7 Alpha-diversity and beta-diversity of plant microbiota calculated using subset community members. Average Shannon indices of 999 times rarefaction calculated from representative ASVs are shown for bacterial (**a**) and fungal (**b**) samples under each condition. Bray-Curtis dissimilarity. PCoA of Bray-Curtis dissimilarities between bacterial (**c**) and fungal (**d**) communities are shown here. R^2 indicates the variance between samples which cannot be explained by compartment, soil type, host species, host genotype and experiment condition.

Supplementary Figures

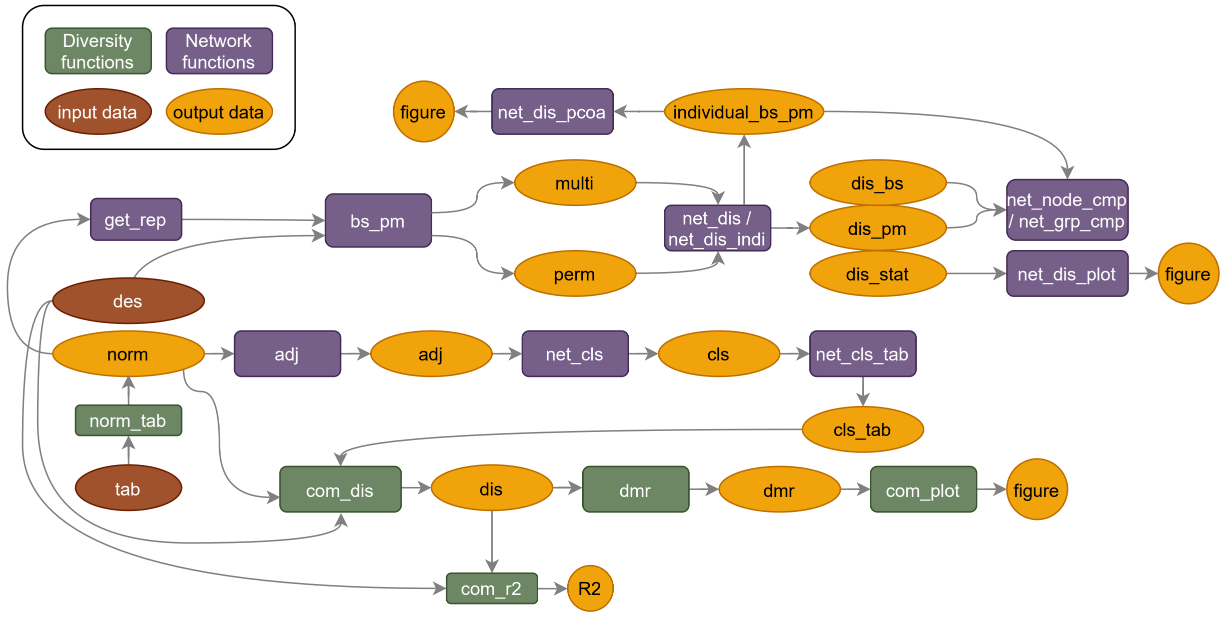

Figure S8 Overview of the R package '*mina*'. Ellipse and rectangles indicate fields and functions of the object '*mina*' respectively. Color indicates different types of functions and data attribute.

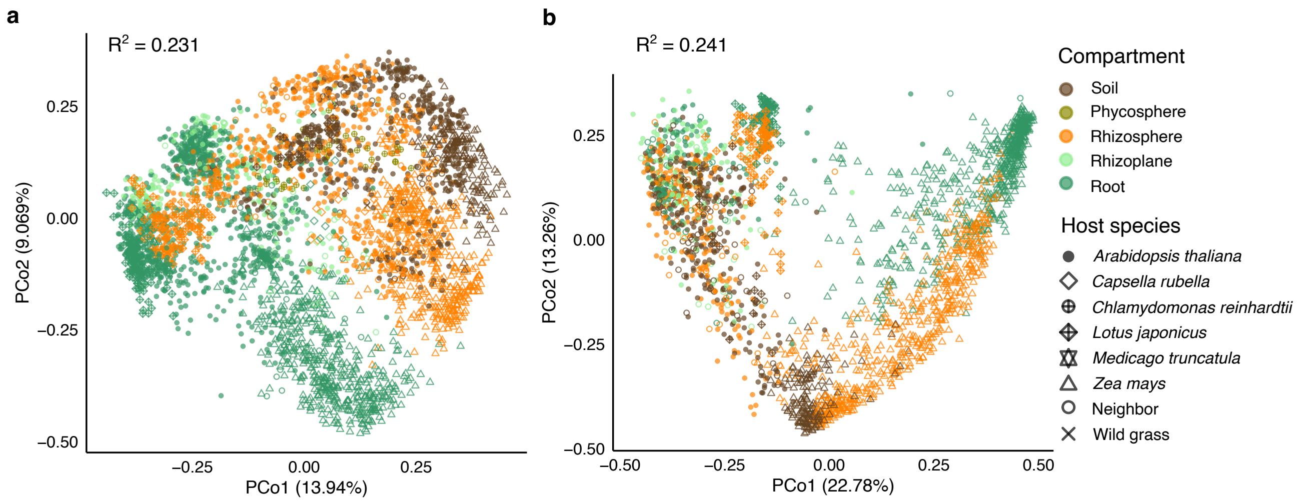

Figure S9 Community diversity of plant-associated microbiota based on repASVs-based network clusters. PCoA of Bray-Curtis dissimilarities between bacterial (**a**) and fungal (**b**) communities are shown here. Network clusters inferred from Spearman correlation matrices (with significant connections only, $P < 0.05$) using AP were used for dissimilarity calculation. R^2 indicates the variance between samples which cannot be explained by compartment, soil type, host species, host genotype and experiment condition.

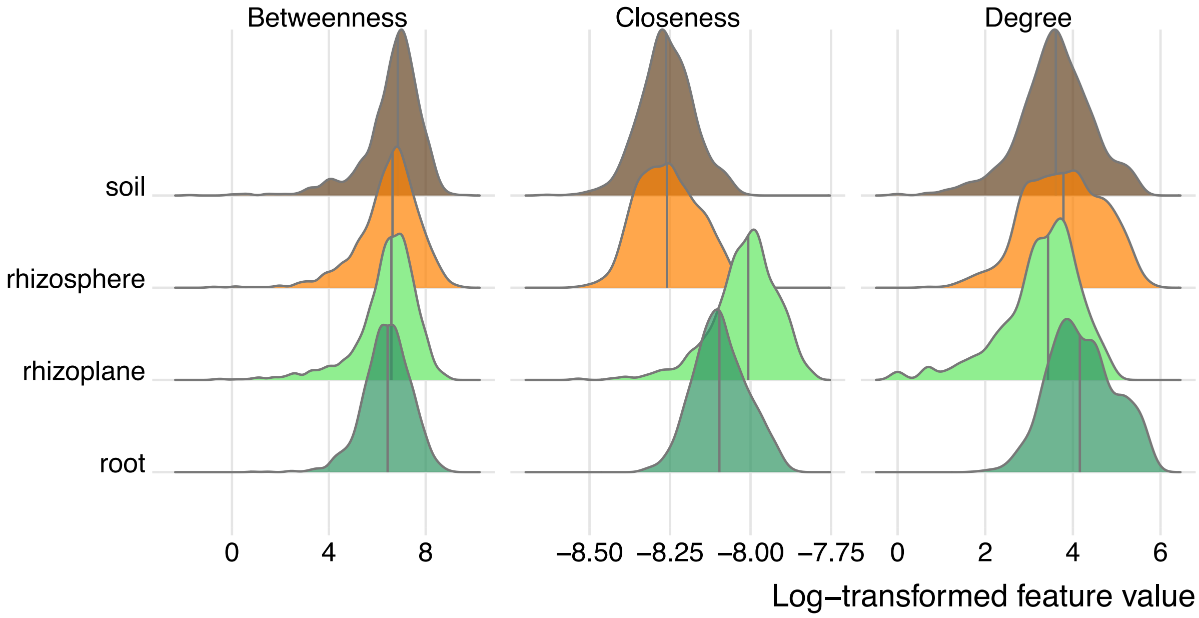

Figure S10 Node betweenness, closeness, and degree distribution of CAS-associated microbial networks.

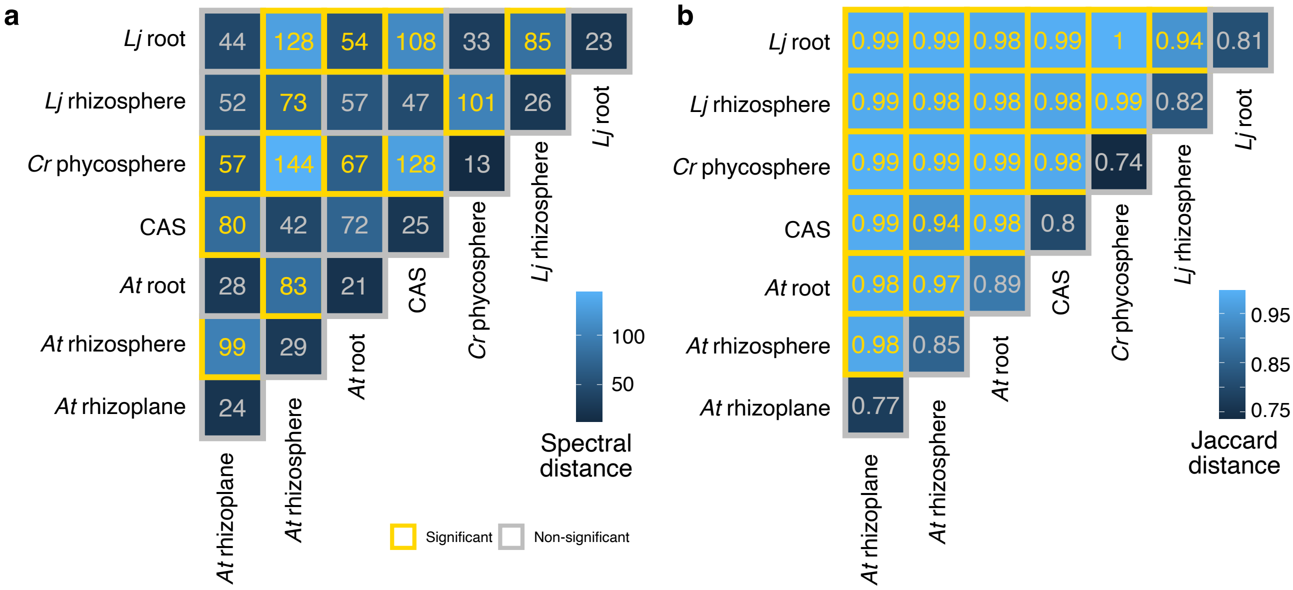

Figure S11 Network distance between different CAS-associated conditions. (**a**) Average of Spectral distance indicating a similar result as observed in Fig. 3a. (**b**) Average of Jaccard distance between networks from different compartments and host species. At: *Arabidopsis thaliana;* Lj: *Lotus japonicus;* Cr*: Chlamydomonas reinhardtii;* CAS: Cologne Agricultural Soil.

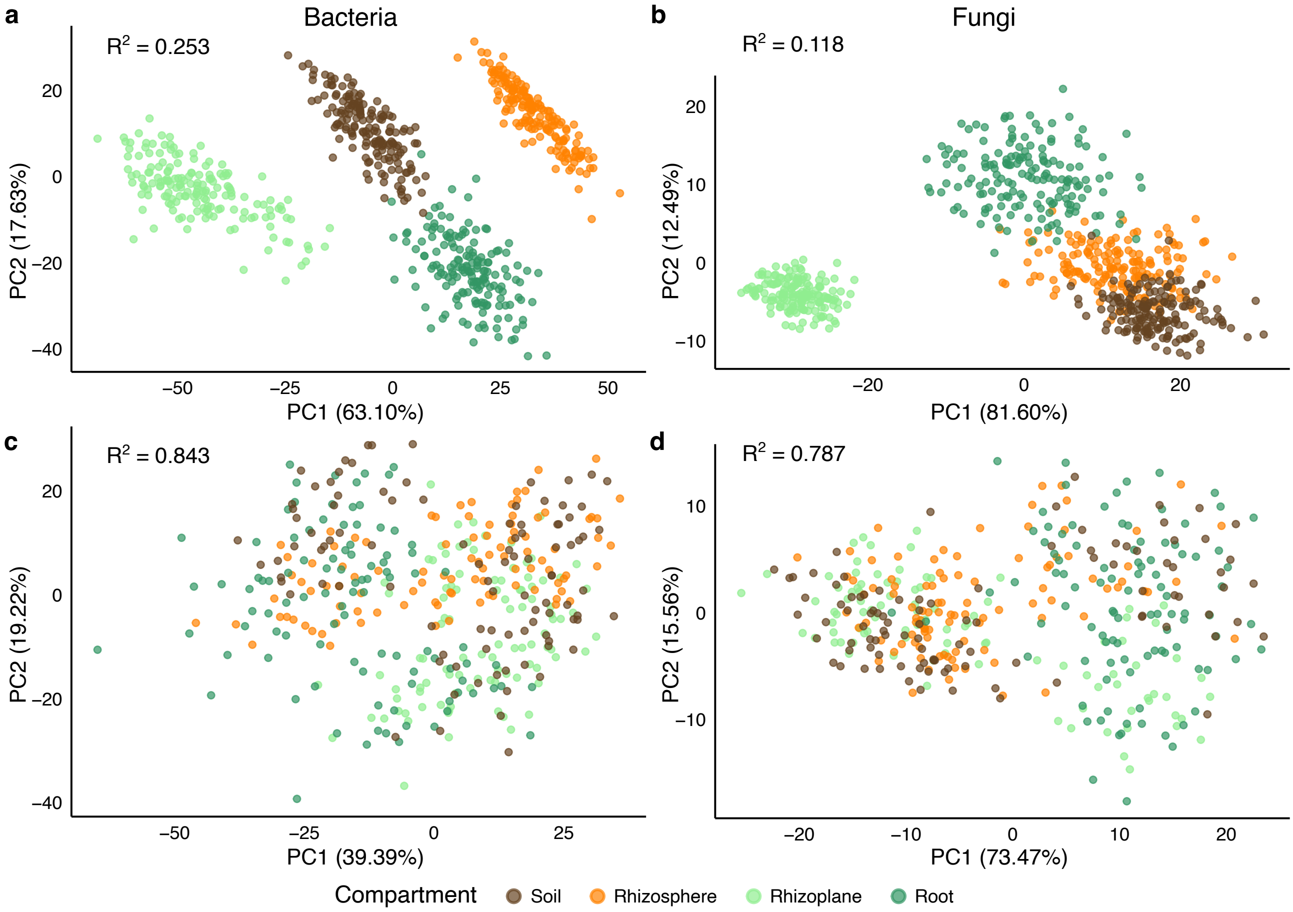

Figure S12 Bootstrap-permutation network analysis of plant-associated microbiota. Observed (**a** bacteria, **b** fungi) and permutated (**c** bacteria, **d** fungi) networks are represented by points. Subsampling time n = 33 for both observed and permutated datasets for both kingdoms.

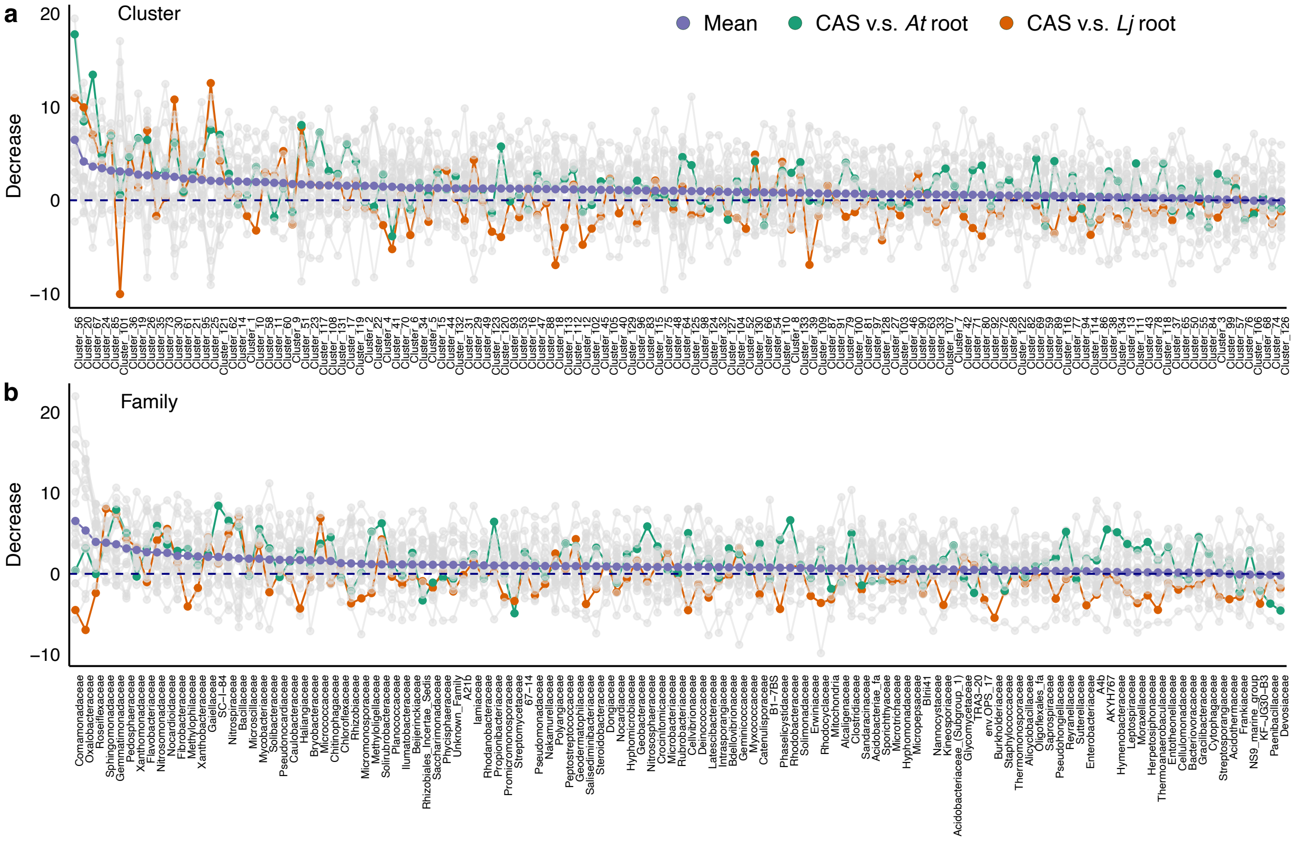

Figure S13 Distance decrease of each permutation group compared to the original network distance. Distance changes caused by each network cluster (**a**) and family (**b**) were sorted by the average decrease of distance between all comparisons (shown in purple). Here we highlighted two soil vs root comparisons: the comparison between CAS and *At* root was shown in green, between CAS and *Lj* root was in orange and other comparisons in grey. At: *Arabidopsis thaliana;* Lj: *Lotus japonicus;* CAS: Cologne Agricultural Soil.

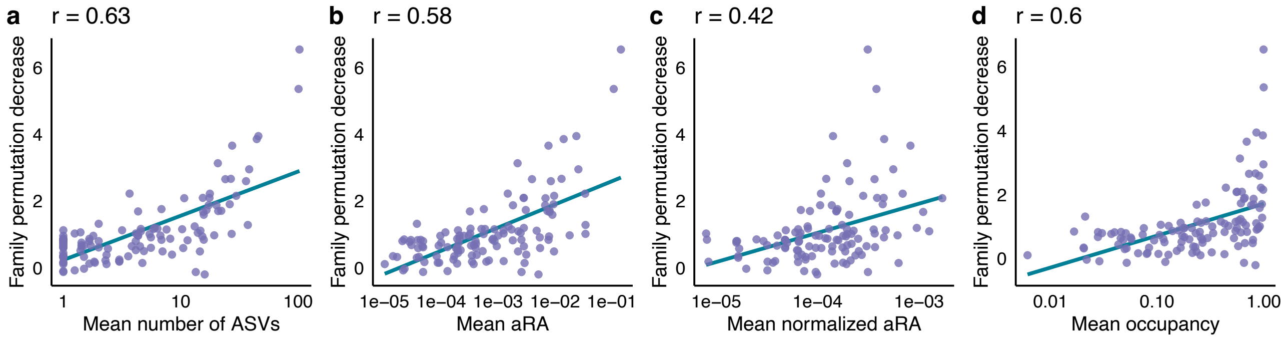

Figure S14 Correlation between permutation distance change and other features of family groups. Y-axis shows the average distance change for each family compared to the distance between bootstrap networks inferred from the original dataset. The teal line is the linear regression of the data points. Spearman correlations are computed, and all coefficients here are significant ($P < 0.001$).

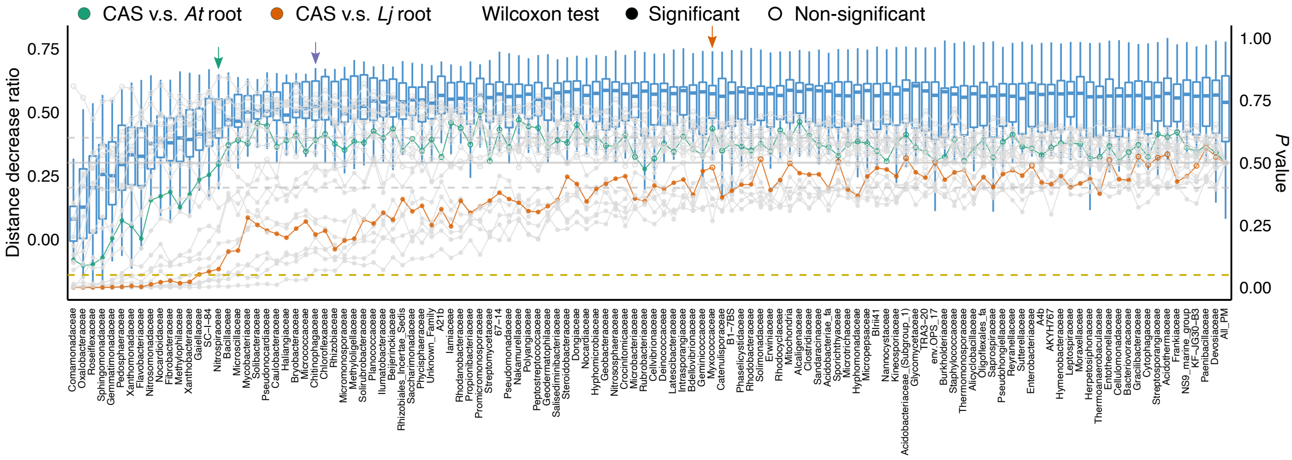

Figure S15 Network distance change for all inter-condition comparisons. Distance change of cumulative group permutation datasets when nodes are grouped by families. Boxplot shows the distance decrease ratio compared to the average distance calculated by bootstrapping the original dataset. The line plot shows the P-value calculated from the permutation test, and the shape of points indicates the significance of the Wilcoxon test with FDR correction. Dash line in gold shows the cutoff of significance ($P = 0.05$).

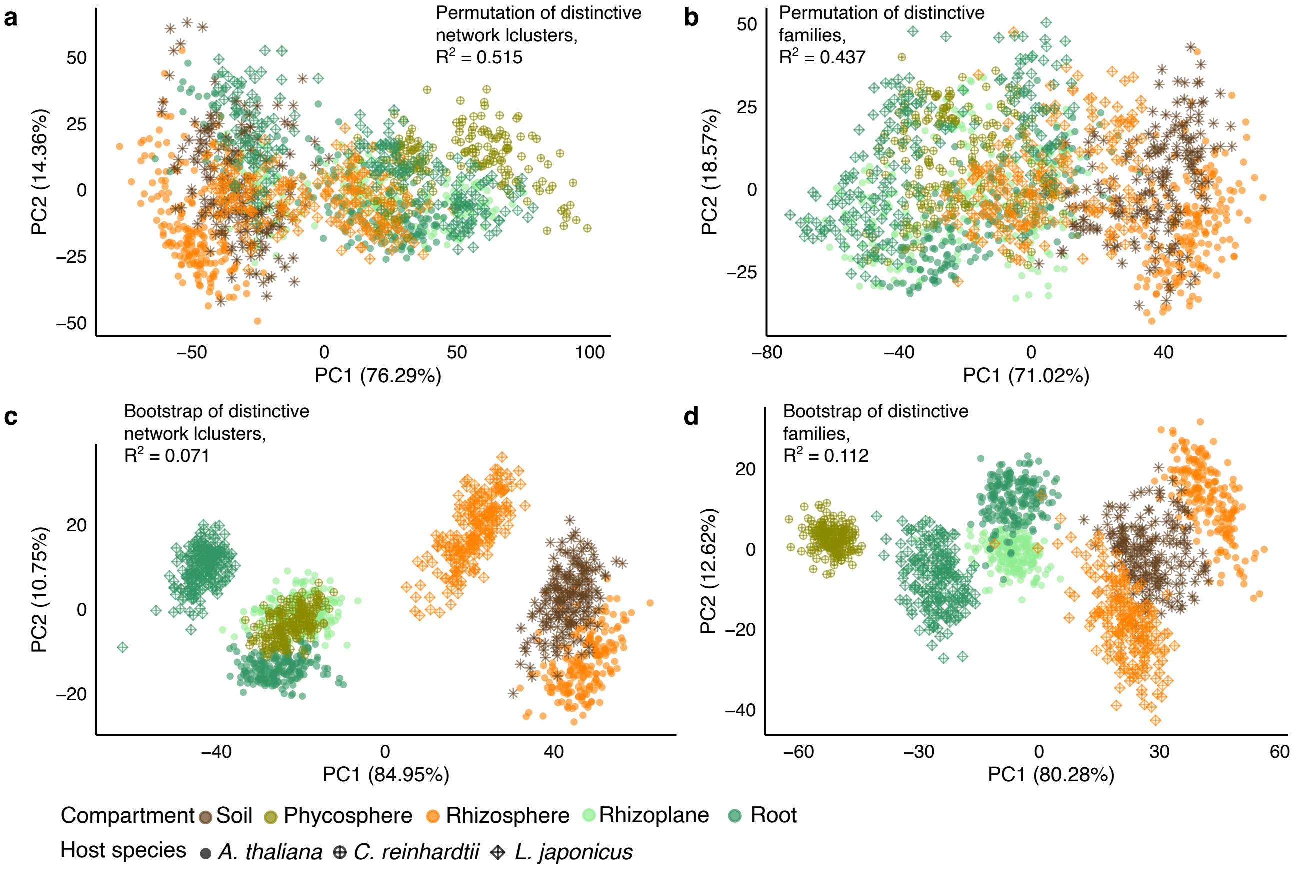

Figure S16 Network comparison between different CAS-associated conditions of distinctive groups. Groups before the distinctive point (n = 26 for both), i.e. family Chitinophagaceae (**a**) and network Cluster_51 (**b**), were accumulatively permutated. For (**c**) and (**d**), only distinctive groups (n = 26 for both) were used for network inference and comparison. PCA of their Spectra distance was shown here. R^2 indicates the variance of Spectra distances between networks, which cannot be explained by compartment and host species.
