# Supplementary material for: Integrated diversity and network analyses reveal drivers of microbiome dynamics
Source: mSystems. 2025 Sep 15;10(10):e00564-25. doi: 10.1128/msystems.00564-25 (PMC12542658; doi:10.1128/msystems.00564-25)
Supplement: Supplemental tables — Table S1 to S6. [file msystems.00564-25-s0002.pdf]

Supplementary Tables

Table S1 Dataset overview. Conditions with less than 30 samples are shown as "Others", including different compartments from host species *Capsella Rubella* and *Medicago truncatula*. Neighbour indicates the neighbouring grass beside the wild *A. thaliana* described in Thiergart *et al.*, 2020.

Host	Compartment	Bacteria	Fungi
Zea mays l	Rhizosphere	498	540
	Root	497	541
Lotus japonicus	Rhizosphere	113	88
	Root	113	78
Chlamydomonas reinhardtii	Phycosphere	48	NA
Arabidopsis thaliana	Rhizosphere	486	186
	Rhizoplane	209	107
	Root	909	101
Neighbor	Rhizosphere	42	44
	Rhizoplane	36	29
	Root	32	42
NA	Soil	760	476
Others	NA	66	NA
Total		3809	2232

Table S2 Number of clusters in bacterial network inferred by Affinity Propagation using different input preference and similarity. Parameters in bold are default values used in '*mina*'.

Parameter *p*	Range of parameter *q*	Min. No. of clusters	Max. No. of clusters
NA	NA	81	81
**NA**	**0**	**81**	**81**
0	0 - 1	81	81
0.1	0 - 1	100	100
0.2	0 - 1	221	221
0.3	0 - 1	609	613
0.4	0 - 1	1222	1231
0.5	0 - 1	1647	1657
0.6	0 - 1	1894	1899
0.7	0 - 1	2001	2004
0.8	0 - 1	2042	2044
0.9	0 - 1	2047	2047
1	0 - 1	2047	2047

Supplementary Tables

Table S3 Number of clusters in bacterial network inferred by Markov Clustering with different expansion and inflation parameters. Bold values are default ones used in '*mina*'.

Expansion	Inflation	No. of clusters
2	2	4
**2**	**2.5**	**4**
2	3	5
2	3.5	6
2	4	9
2	4.5	9
2	5	9
2.5	2	4
2.5	2.5	4
2.5	3	5
2.5	3.5	6
2.5	4	9
2.5	4.5	9
2.5	5	9
3	2	2
3	2.5	3
3	3	4
3	3.5	4
3	4	4
3	4.5	4
3	5	4
3.5	2	2
3.5	2.5	3
3.5	3	4
3.5	3.5	4
3.5	4	4
3.5	4.5	4
3.5	5	4
4	2	1
4	2.5	2
4	3	3
4	3.5	3
4	4	3
4	4.5	3
4	5	4
4.5	2	1
4.5	2.5	2
4.5	3	3
4.5	3.5	3
4.5	4	3
4.5	4.5	3
4.5	5	4
5	2	1
5	2.5	1
5	3	2
5	3.5	2
5	4	3
5	4.5	3
5	5	3

Supplementary Tables

Table S4 Unexplained variance ratio of microbial communities. Diversity analyses based on ASVs and network clusters were shown here.

Network	Clustering	Bacteria Number of taxa	Bacteria Unexplained ratio (%)	Fungi Number of taxa	Fungi Unexplained ratio (%)
/	ASVs	42,060	51.3	9337	46.0
/	repASVs	2047	43.1	370	39.0
Pearson	MCL	45	15.7	36	26.2
	AP	191	29.4	58	31.5
Spearman	MCL	4	10.6	4	7.6
	AP	81	23.1	21	24.1
SparCC	MCL	3	9.3	2	10.7
	AP	78	23.1	29	23.6

Table S5 Sample number of Cologne Agricultural Soil-derived conditions.

Hose Species	Compartment	Sample
	Soil	86
C. reinhardtii	Phycosphere	48
L. japonicus	Rhizosphere	113
	Root	113
A. thaliana	Rhizosphere	133
	Rhizoplane	62
	Root	299

Table S6 Network features of CAS-associated microbiota.

Compartment	No. of nodes	No. of edges	No. of positive edges	No. of negative edges	Density
Soil	1477	37,401	30,768	6633	0.03
Rhizosphere	1572	50,040	42,102	7938	0.04
Rhizoplane	1082	19,552	18,254	1298	0.03
Root	1481	66,518	52,294	14,224	0.06
